# Alemtuzumab-Related Lymphocyte Subset Dynamics and Disease Activity or Autoimmune Adverse Events: Real-World Evidence

**DOI:** 10.3390/jcm12051768

**Published:** 2023-02-22

**Authors:** Elisabetta Signoriello, Giacomo Lus, Francesco Saccà, Marco Puthenparampil, Cinzia Coppola, Andrea Di Pietro, Gianfranco Puoti, Maria Cristina Criscuolo, Matteo Foschi, Giuseppina Miele, Gianmarco Abbadessa, Vincenzo Brescia Morra, Paolo Gallo, Simona Bonavita, Maria Pia Sormani, Alessio Signori

**Affiliations:** 1 Multiple Sclerosis Center, Second Division of Neurology, Department of Advanced Medical and Surgical Science, University of Campania Luigi Vanvitelli, 80131 Naples, Italy; 2 Department of Neurosciences, Reproductive and Odontostomatological Sciences, Federico II University, 80131 Naples, Italy; 3 Multiple Sclerosis Center, Department of Neuroscience, Università degli Studi di Padova, 35122 Padova, Italy; 4 Department of Neuroscience, Multiple Sclerosis Center—Neurology Unit, S. Maria delle Croci Hospital of Ravenna, AUSL Romagna, 48121 Ravenna, Italy; 5 Department of Medical and Surgical Sciences, University of Bologna, 40126 Bologna, Italy; 6 Department of Health Sciences, University of Genova, 16132 Genova, Italy

**Keywords:** multiple sclerosis, alemtuzumab, induction treatment, lymphocyte subsets

## Abstract

Background and objectives: alemtuzumab is a monoclonal anti-CD52 antibody acting on B and T cells in highly active multiple sclerosis (MS). We analyzed changes in lymphocyte subsets after alemtuzumab administration in relation to disease activity and autoimmune adverse events. Methods: lymphocyte subset counts were assessed longitudinally using linear mixed models. Subset counts at baseline and during follow-up were correlated with relapse rate, adverse events, or magnetic resonance (MRI) activity. Results: we recruited 150 patients followed for a median of 2.7 years (IQR: 1.9–3.7). Total lymphocytes, CD4, CD8, and CD20 significantly decreased in all patients over 2 years (*p* < 0.001). Previous treatment with fingolimod increased the risk of disease activity and adverse events (*p* = 0.029). We found a higher probability of disease reactivation in males and in patients with over three active lesions at baseline. Higher EDSS scores at baseline and longer disease duration predicted the switch to other treatments after alemtuzumab. Discussion and conclusions: Our real-world study supports data from clinical trials in which lymphocyte subsets were not useful for predicting disease activity or autoimmune disease during treatment. The early use of an induction therapy such as alemtuzumab in patients with a lower EDSS score and short history of disease could mitigate the risk of treatment failure.

## 1. Introduction

Alemtuzumab is a humanized monoclonal antibody directed against the CD52 antigen (a small glycoprotein on the surface of mature T and B lymphocytes) and to a lesser extent, myeloid cell types [1]; it is not expressed on stem/progenitor immune cells, erythrocytes, or platelets. CD52 is essential for lymphocyte transendothelial migration and may contribute to costimulation of CD4^+^ T cells and T cell activation and proliferation. It represents one of the most powerful disease-modifying therapies for relapsing–remitting multiple sclerosis (MS) patients and can induce a long-term remission after only two cycles of treatment [2]. Alemtuzumab is classified as an immune reconstitution therapy (IRT), as is autologous hematopoietic stem cell transplantation (AHSCT). After a profound short-term immune suppression, the immune system undergoes radical changes leading to a persistent immune tolerance [3]. Each treatment cycle induces a rapid and deep pan-lymphocyte depletion and more subtle effects on NK cells, neutrophils, monocytes, and dendritic cells. Then, every cell type recovers with a different speed: T cell lymphopenia is prolonged, as CD4 and CD8 reach the lower limit of normality after an average of about 35 and 20 months, respectively. B-lymphocytes reach reference levels within 6 months [4].

Despite its high efficacy, the use of alemtuzumab is limited by the risk of opportunistic infections and secondary autoimmune disorders. Immune reconstitution is responsible for secondary autoimmune diseases and has an incidence peak from 2 to 3 years after treatment administration. In particular, B-cell autoimmune disorders are more frequent and consist of antibody-mediated thyroid disease, idiopathic thrombocytopenic purpura, and anti-glomerular basement membrane (GBM) disease. T cell-mediated autoimmunity and granulomatous inflammatory diseases are far less frequent [5].

Over the 12-year follow-up of the CAMMS223 trial, 73% of patients were free from MRI disease activity, 71% of patients had stable or improved Expanded Disability Status Scale (EDSS) scores, and 69% were free from 6-month confirmed disability worsening. Cumulatively throughout the extensions (years 7–12), 34% of patients had no evidence of disease activity (NEDA). Adverse event (AE) incidence declined through year 12 [6].

The identification of a biomarker that predicts the risk of recurrent disease activity or the development of secondary autoimmune disorders is desirable. In this multicenter study, we aimed to describe lymphocyte dynamics after alemtuzumab treatment and their correlation with disease recurrence and autoimmunity.

## 2. Methods

This was a retrospective analysis of data collected from 4 Italian MS centers. We included all MS patients diagnosed according to the 2010 revisions of the McDonald Criteria [7] who started alemtuzumab treatment according to clinical practice between 2015 and 2019.

For each patient, we collected demographic data (age, sex), MS course (relapsing vs. progressive), MS history (MS onset date, age at onset, disease modifying therapies (DMTs) before alemtuzumab); date and number of alemtuzumab infusions; clinical and instrumental (MRI) activity before alemtuzumab treatment and during follow-up (number of relapses during the year before and after alemtuzumab infusion, gadolinium-enhancing lesions at baseline MRI and every six months after infusion, MS disability with expanded disability status scale (EDSS) when starting alemtuzumab and every 6 months after treatment). We collected data on absolute lymphocyte counts and subsets (i.e., CD20, CD4, CD8, and NK counts) before and after the initiation of alemtuzumab.

This study was conducted in accordance with the ethical standards of the Declaration of Helsinki. Given its observational design, the sample size was not assessed.

### Statistical Analysis

Mean with standard deviation (SD) or median with interquartile range (IQR) or range were reported for continuous variables.

A complete-case analysis was conducted at each time point and main results were reported without imputation of missing data. However, the last available data within 24 months of follow-up of each lymphocyte subset was also reported and difference from baseline was calculated.

A linear mixed regression model (LMM) was used to statistically test the longitudinal trend of lymphocyte subsets used as dependent variables. The interaction test with time was used for each outcome (i.e.: MRI activity or relapse) to test if patients with and without each outcome had a different longitudinal shape of lymphocyte subsets over 24 months. For the LMM, a logarithmic transformation of each lymphocyte subset was adopted before their execution due to the skewed distribution.

A logistic regression model was used to assess the impact of baseline lymphocyte subsets and previous DMTs (fingolimod, natalizumab, and other DMTs) on binary outcomes (i.e., MRI and relapse activity and composite outcome with adverse events).

For all the previous analyses, the standardized mean difference (SMD) between the compared groups was reported as effect size for the continuous and binary features.

The Kaplan–Meier (KM) method was used to estimate the time to switch after alemtuzumab, while Cox regression analysis was used to define the association of clinical characteristics and lymphocyte subsets to the time to switch. Results were reported as hazard ratios (HRs) with a corresponding 95% confidence interval (CI).

Stata (v.16; StataCorp., College Station, TX, USA) was used for the computation.

## 3. Results

We included 150 patients; demographic and clinical characteristics at alemtuzumab start are summarized in Table 1. Median follow-up after alemtuzumab start was 2.7 years (IQR: 1.9–3.7). Relapse rate before alemtuzumab start was 0.69 (SD: 0.92) and decreased to 0.11 (SD 0.34) during the first year of treatment, 0.10 (SD 0.29) after 2 years, and 0.082 (SD 0.22) for the entire follow-up. We found 15 patients (10%) with at least one clinical relapse after the first year of treatment, 22 (14.7%) over two years, and 24 patients for the entire follow-up (16%). We found 25 patients with gadolinium-enhancing lesions or with an increase in lesion load in the MRI during the first year of follow-up. The number increased to 35 during the second year. Globally, 32 (21.3%) patients in the first year and 44 patients (29.3%) during the first two years had clinical or instrumental relapses. We report a total of 62 adverse events (41.3%), including 22 (14.7%) of autoimmune origin, in particular thyroiditis (n = 18). Among others, we observed cutaneous rush (n = 16), bradycardia (n = 12), and headache (n = 8).

### 3.1. Lymphocytic Dynamics and Disease Activity or Autoimmune Adverse Events in the First Two Years of Treatment

#### 3.1.1. Lymphocytes

A total of 110 patients (73.3%) had available lymphocyte data for at least two time points. Mean total lymphocytes were 2241 (SD: 1160; median: 1995; n = 110) at baseline. Sixty-six patients had available data both at baseline and at 6 months, with a median decrease of 1075 (IQR: −2362, −410) while 82 patients had both baseline and 12 months, with a median decrease of 913 (IQR: −1800, −260) and 56 patients had baseline and 24 months and a median decrease of 640 (IQR: −1615, −35) from baseline. Considering the last available data over 24 months (n = 106), the decrease was −885 (IQR: −1850, −240).

A significant difference compared with baseline was registered at all timepoints (*p* < 0.001). No significant differences were observed between months 12 and 24 (*p* = 0.35), as well as between months 6 and 18 (*p* = 0.94), while the decrease was significant between months 6 and 12 (*p* = 0.038).

**Figure 1:**
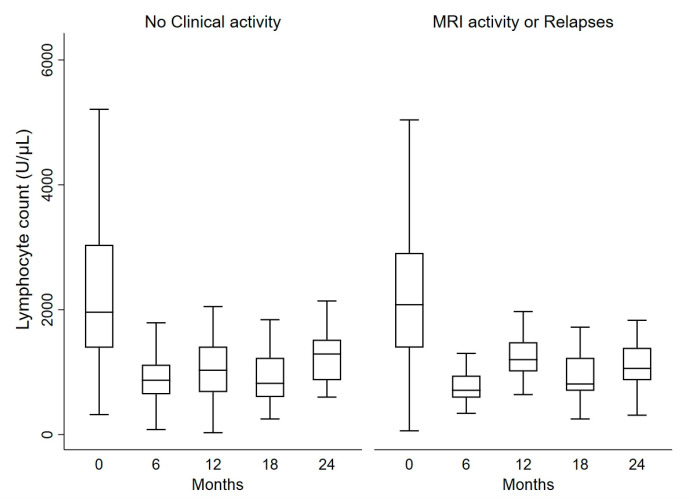
Lymphocyte dynamics in patients with and without clinical or instrumental activity. Clinical activity means the occurrence of disease relapses and instrumental activity means occurrence of gadolinium-enhancing lesions in MRI.

No differences in lymphocyte levels emerged when comparing patients with or without relapses after 1 year of alemtuzumab (*p* = 0.83) or over 2 years (*p* = 0.37). The same was true regarding MRI activity after 1 (*p* = 0.73) or 2 years (*p* = 0.45). We found a significant difference in lymphocyte counts for the composite outcome of MRI activity and clinical relapses after 2 years of alemtuzumab (*p* = 0.020): at 12 months the lymphocyte decrease in the group without MRI activity or relapses (n = 60; median decrease: −1005; IQR: −2000, −304) was more consistent as compared with active patients (n = 22; median decrease: −785; IQR: −1600, −80) (Figure 1). Similar results were observed in terms of MRI activity and relapses only over the first year (median difference: −970 in not active vs. −790 in active patients).

Baseline lymphocyte count was not associated with any of the previously stated outcomes.

#### 3.1.2. CD8

The CD8 subset count in at least two time points was available for 89 patients and showed a similar trend to total lymphocytes. At baseline, CD8 had a mean value of 458 (SD: 276; median: 382; n = 89). Fifty-two patients had both baseline and 6-month data, with a median decrease of 190 (IQR: −472, −74), while the decrease at 12 months was 127 (IQR: −344, −34; n = 61). In 44 patients with available data, the decrease at 24 months was 150 (IQR: −286, −37). Considering the last available data over 24 months (n = 85), the decrease was 180 (IQR: −377, −50).

All timepoints were significantly different from baseline (*p* < 0.001). We found no difference between 12 and 24 months (*p* = 0.21) and between 6 and 18 months (*p* = 0.74), but CD8 levels were significantly lower at 6 months compared to 12 months (*p* = 0.002). There was no difference in CD8 levels when comparing patients with or without composite outcome of MRI activity and clinical relapse (*p* = 0.31) or comparing patients with adverse events (*p* = 0.41).

#### 3.1.3. CD4

The CD4 count was available in at least two time points for the same patient except one (n = 88). Mean baseline value was 835 (SD: 303; median: 704). CD4 decreased by 422 units (IQR: −1014, −225) at 6 months, by 382 units (IQR: −1019, −200) at 12 months, and by 320 (IQR: −817, −151) at 24 months, with a significant difference at all timepoints as compared with baseline (*p* < 0.001). Considering the last available data over 24 months (n = 85), the decrease was 180 (IQR: −377, −50).

We found no differences in CD4 counts when comparing patients with a positive outcome of MRI activity and clinical relapse (*p* = 0.94 over 1 year and *p* = 0.22 over 2 years) or on the global composite outcome (*p* = 0.57). There was no association between baseline values and any of the previously stated outcomes (composite outcome over 2 years: *p* = 0.76).

#### 3.1.4. CD20

The CD20 count at baseline was available for 77 patients. Mean baseline value was 327 (SD: 280; median: 242) and was substantially stable at 6 (median difference: 29; IQR: −140, 180; n = 39), 12 (median difference: 14; IQR: −120, 150; n = 46) and 24 months (median difference: 30; IQR: −120, 150; n = 30). The time effect was a trend (*p* = 0.062).

Considering the last available data over the 24 months, the median difference from baseline was −15 (IQR: −175, 102).

We found no impact of CD20 levels on the composite outcome with MRI activity and clinical relapses after 1 (*p* = 0.93) or 2 years (*p* = 0.72) and for the composite outcome with adverse events over 2 years (*p* = 0.65). Higher baseline values resulted in a trend for a higher probability of autoimmune adverse events (OR = 3.72, 95% CI: 0.92–15.0; *p* = 0.065) and for a lower probability of MRI activity over 1 year (OR = 0.12; 95% CI: 0.01–1.06; *p* = 0.056).

#### 3.1.5. NK

The NK count for at least two time points was available for 46 patients and at baseline had a mean value of 184 (SD: 149; median: 136). Patients showed a significant (*p* = 0.016) increase over time. At 6 months, the median increase was 103 (IQR: −11, 165; n = 28), at 12 months 60 (IQR: 0–130; n = 35), and at 24 months 66 (IQR: 18–140; n = 22). Considering the last available data over the 24 months, the change from baseline was 55 (IQR: −5, 134).

**Figure 2:**
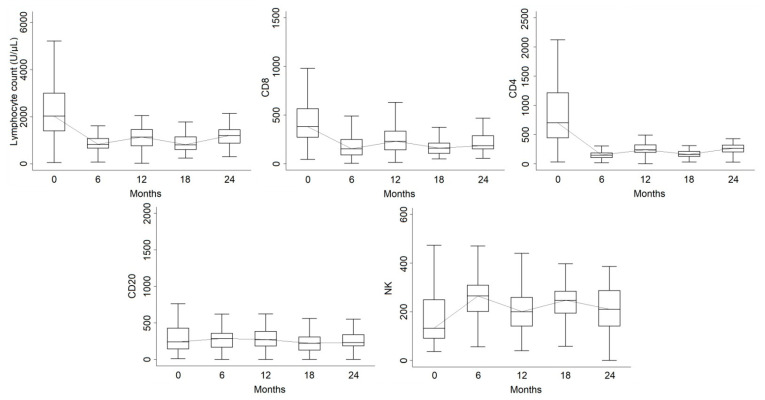
Boxplot of lymphocyte subset longitudinal trends over 24 months. The line represents the longitudinal trend of the median value for each subset reported.

No differences emerged for longitudinal changes in NK nor comparing patients on composite outcome of MRI activity or clinical relapse (*p* = 0.46 over 1 year and *p* = 0.26 over 2 years) nor on global composite outcome (*p* = 0.97). We found no association between baseline values and any of the previously stated outcomes (composite outcome over 2 years: *p* = 0.60). The lymphocyte subset longitudinal trend over 24 months is shown in Figure 2.

### 3.2. Previous Treatments and Their Impact on Lymphocyte Subsets

We analyzed the lymphocyte subset trends on the basis of previous treatments. For this analysis, the last available value of lymphocyte subsets over 24 months was used.

At the baseline, patients previously treated with natalizumab, fingolimod, or other DMTs had significantly different lymphocyte counts (*p* < 0.001 for all three comparisons). This was not confirmed 24 months after alemtuzumab treatment (global *p*-value: 0.51).

**Figure 3:**
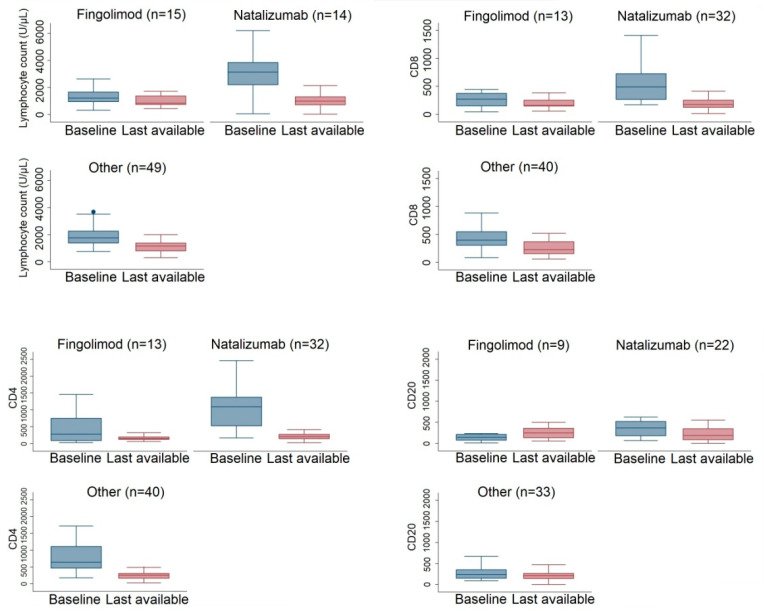
Boxplot of lymphocyte subsets stratified for previous DMTs (fingolimod, natalizumab, or others) at baseline and at the last available observation within 24 months. Data at 24 months were used if available, otherwise 18 months or 12 months were considered.

Indeed, during the 24 months, we found median lymphocyte change was −249 (IQR: −1040, 350) for fingolimod vs. −1920 (IQR: −2940, −880) for natalizumab and −746 (IQR: −1120, −175) for other DMTs (Figure 3). CD8 levels at baseline were not different between natalizumab and other DMTs (*p* = 0.60) but both had higher values compared with fingolimod (*p* = 0.0072 Nat; *p* = 0.0049 vs. other DMTs). After 24 months, we observed no significant differences in CD8 levels (global *p*-value: 0.29) (Figure 3). Baseline CD4 levels were not different between natalizumab and other DMTs (*p* = 0.091) but both showed higher values as compared to fingolimod (*p* = 0.001 Nat; *p* = 0.0095 vs. other DMTs). No significant differences were found during alemtuzumab treatment (global *p*-value: 0.10). Similarly, CD20 levels were similar between natalizumab and other DMTs (*p* = 0.11) but higher than fingolimod (*p* = 0.022 Nat; *p* = 0.046 vs. other DMTs). CD20 levels were similar between groups during alemtuzumab treatment (global *p*-value: 0.86). We found a median increase of 130 (IQR: −93, 170) for fingolimod vs. a difference of −78 (IQR: −303, 23) for natalizumab and −2 (IQR: −83, 103) for other DMTs (Figure 3).

Although a higher probability of MRI activity or clinical relapses in the first year was observed for patients switched from fingolimod (OR = 2.56 (95% CI: 0.61–10.75); *p* = 0.20) or natalizumab (OR = 2.58 (95% CI: 0.66–10.18); *p* = 0.18), statistical significance was not reached. Similar results were observed over two years of follow-up. For composite outcomes with adverse events, we observed a higher probability as compared to naive patients for patients switched from fingolimod (OR = 3.86; 95% CI: 1.15–12.92; *p* = 0.029), while for natalizumab statistical significance was not reached (OR = 2.76; 95% CI: 0.87–8.74; *p* = 0.084).

### 3.3. Lymphocytic Dynamics Do Not Predict Disease Reactivation after 24 Months of Treatment

Total median follow-up after alemtuzumab treatment was 2.7 years (IQR: 1.9–3.7), with 62 patients not meeting the 2-year time point. Sixteen patients had a clinical relapse or evidence of MRI activity during the first 2 years of alemtuzumab treatment. Table 2 reports clinical and radiological features of patients with clinical or MRI activity after 2 years vs. those without activity but with follow-up >2.5 years. We found a higher probability of clinical relapse or MRI activity in male patients and in patients with active lesions at baseline, especially in those with more than three gadolinium-enhancing lesions. Upon examining the group of patients with or without activity after 24 months independently from length of follow-up, these results were confirmed (Table S1).

### 3.4. Time to Switch to Other Treatments after Alemtuzumab

A total of 24 patients (16%) switched to another treatment. The main switch was to ocrelizumab (n = 15), followed by fingolimod (n = 5), rituximab (n = 3) and HSCT (n = 1).

KM estimates of switch after 2 and 4 years from the first dose were, respectively, 5.8% (95% CI: 2.8–11.9%) and 28.9% (95% CI: 19.4–41.7).

In Table 3 clinical characteristics split by switchers and no-switchers are reported. Patients with a higher disease duration and worse baseline EDSS switched earlier to another treatment.

## 4. Discussion

Alemtuzumab represents a highly effective therapy in MS patients, either active naive or switching from first- and second-line therapies. From clinical trials and real-world studies, long-term complete control of disease activity is detectable in a high percentage of patients, although in the first or second year of treatment some patients might be suboptimal responders with an occurrence of clinical or MRI activity. Furthermore, it remains to be determined whether early disease activity during induction treatment could predict its failure [8].

In our study, we recorded a clinical or radiological relapse in 21.3% of patients during the first year and 29.3% over 2 years. These results aligned with findings from the recent large real-world study by Russo et al., in which 28.4% and 41.1% of patients showed MRI activity, relapses, or EDSS score progression at 1- and 2-year follow up, respectively [9]. The percentage of suboptimal responders seems to increase during the long-term follow up [10], thus suggesting lower efficacy, at least in a subgroup of patients. However, clinical or biological metrics associated with a higher risk of disease reactivation are currently lacking. In this regard, Baker et al. recently suggested the detection of anti-alemtuzumab antibodies as a useful biomarker to identify patients resistant to treatment [11]. Regarding adverse events, we reported a frequency of 41.3% global adverse events, including 14.7% with autoimmune origin. This result was lower compared to prior studies, probably due to the shorter duration of our follow-up [1].

After alemtuzumab-induced depletion mediated by the CD52 antigen, immune reconstitution follows different trajectories according to cell type. B cells recover rapidly, whereas T cell depletion is prolonged, with CD4 and CD8 cells taking years to reach the lower limit of normal [11]. This supports a relationship between different lymphocyte dynamics and the development of autoimmune adverse events, especially thyroiditis.

In our study, as is imaginable, the trend of global lymphocytes and subpopulations showed a decrease at 6 and 18 months after treatment, with a subsequent increase at 12 and 24 months, returning to lower values than at the baseline [12]. In particular, we observed in our patients a decrease in global lymphocytes and the CD4 and CD8 subsets at all time points, while we recorded a stationary count of B cells with increased NK counts over time. This observation is in accordance with the lymphocyte dynamics reported in other studies [13] and in particular with a recent study by Palmieri et al., in which the increase in NK cells seems to influence reconstitution of T and B cells in an anti-inflammatory profile [14]. Looking at the baseline immune constitution of our patients, we observed a trend of lower disease activity and a higher probability of autoimmune adverse events in patients with higher CD20 counts at baseline. It can be speculated that this finding may reflect a strong effect of alemtuzumab on this subpopulation, leading to both control of disease activity and to the regeneration of immature B lymphocytes associated with autoimmune events [13]. Patients without relapses at 12 months had a higher lymphocyte decrease compared to patients with MRI activity or relapses, probably due to more consistent immune depletion.

In our population, lymphocyte baseline counts were statistically different according to the type of DMT before alemtuzumab. Prior treatment with fingolimod (a sequestering agent) was associated with lower levels of lymphocytes at baseline, potentially hampering the early effectiveness of alemtuzumab, as demonstrated for other depleting therapies (e.g., ocrelizumab) [15]. In our study, switching from fingolimod to alemtuzumab was related to a higher probability of disease activity and adverse events, supporting observations in recent reports [16,17]. This phenomenon could also be explained by the specific profile of circulating lymphocytes at the withdrawal of fingolimod, which can foster an increase in T and B regulatory subsets [18,19].

It is of interest that during therapy there was a similar reduction over time of lymphocytes and subpopulations regardless of their baseline value or previous treatment, unlike other depletive agents, which can induce a chronic reduction of T lymphocytes (e.g., ocrelizumab after fingolimod) [20].

Regarding predictors of disease reactivation during follow-up, we saw a higher risk of disease reactivation in males and patients with more than three gadolinium-enhancing lesions at basal MRI, whereas total and subset lymphocyte dynamics did not predict the occurrence of clinical or instrumental relapses. This observation was in contrast with a prior report [21] including 56 patients treated with alemtuzumab, suggesting that peripheral CD4 recovery could be a biomarker of disease activity after treatment. Specifically, the authors suggested a specific CD4 cutoff (greater than 388.5 × 10^6^ cells/mL at 12 months), identifying patients who were likely to have recurrent disease activity [21]. Furthermore, in line with a study by Jones et al. [22], we confirmed that neither CD4 nor CD20 rebound is associated with a recurrence in disease activity or autoimmune adverse events. Indeed, in this cohort of 108 patients with long-term follow-up, no significant difference was recorded in the recovery of any cell population between patients with and without disease activity or accumulation of disability after alemtuzumab treatment [22]. We obtained these results with a larger cohort of patients compared to other studies and with a greater number of disease activity events, which increases the statistical power of the analysis.

Additionally, we analyzed patients who switched to other DMTs after alemtuzumab. Non-responders were identified on a clinical basis. Patients with longer disease duration and higher EDSS scores at baseline were at a higher risk of switching to other DMTs after alemtuzumab, possibly due to the delay in the initiation of induction treatment. Annualized relapse rates and MRI activity during the first two years of treatment did not increase the risk of switching. Therefore, early suboptimal response did not enhance disease reactivation nor predict a switch to another DMT. At baseline, higher levels of lymphocytes and the CD4 and CD8 subsets were predictors of treatment switch, perhaps reflecting a higher degree of disease activity [23].

Limitations of our study included the retrospective design, the lack of a standardized protocol for lymphocyte measurements at scheduled time points, and the relatively short follow-up.

On the other hand, this was a multicenter real-world study exploring lymphocytes subsets with the largest number of patients to date.

In conclusion, the early use of an induction therapy such as alemtuzumab in patients with lower EDSS scores and short disease duration could mitigate the risk of treatment failure. In clinical practice, lymphocyte subset dynamics do not help in identifying patients at risk of disease reactivation; furthermore, the CD20 subset during the follow-up does not seem to be useful in identifying patients at risk of autoimmune adverse events.

## Figures and Tables

**Table 1:** Demographic and clinical characteristics of the included cohort.

	n = 150
**Age**, mean (SD), range	36 (10.4), 18–66
**Females**, n (%)	103 (68.7)
**Years from symptom onset**, median (IQR)	6.7 (2.5–12.5)
**Years from diagnosis**, median (IQR)	5.1 (2.0–10.3)
**Baseline EDSS**, median (IQR), range	3 (2–5), 0–8.5
**ARR previous year**, mean (SD)	0.69 (0.92)
**Active lesions at baseline**, n (%)	
0	63/136 (46.3)
1	24/136 (17.7)
2	20/136 (14.7)
3+	29/136 (21.3)
N. of previous DMTs, median (range)	2 (0–6)
**Previous DMTs**, n (%)	
Naive	23 (15.3)
Natalizumab	50 (33.3)
Fingolimod	33 (22.0)
Dimethyl-fumarate	21 (14.0)
GA	9 (6.0)
Interferon	7 (4.7)
Other	7 (4.7)
**Wash-out**, months median (IQR)	2 (1.3–3.4)
**Follow-up**, years median (IQR)	2.7 (1.9–3.7)
**N. of cycles**, n (%)	
1	12 (8)
2	135 (90)
3	3 (2)

**Table 2:** Clinical characteristics in patients with and without clinical or MRI activity after 24 months from treatment start.

	Clinical or MRI Activity after 24 Months (n = 16)	No Clinical Activity after 24 Months (n = 72)	SMD	*p*-Value
**Age**, mean (SD), range	32.5 (9.4), 18–56	36.2 (10.0), 18–63	0.38	0.16
**Females**, n (%)	7 (43.8)	54 (75)	0.66	0.020
**Years from symptom onset**, median (IQR)	6.3 (2.5–11.1)	8.0 (3.2–13.3)	0.17	0.47
**Years from diagnosis**, median (IQR)	4.7 (2.1–10.3)	5.4 (2.5–10.9)	0.10	0.62
**Baseline EDSS**, median (IQR), range	2.5 (1–6), 1–7	3.5 (2–5.5), 1–8.5	0.22	0.39
**Wash-out months before lemtrada**, median (IQR)	1.5 (1.1–2.4)	1.8 (1–3.4)	0.01	0.92
**N. of previous DMTs**, median (range)	2 (0–6)	2 (0–6)	0.00	0.98
**Previous DMTs**, n (%)			NC	0.93
Naive	2 (12.5)	10 (13.9)		
Natalizumab	4 (25)	22 (30.6)		
Fingolimod	5 (31.3)	15 (20.8)		
Dimethyl-fumarate	3 (18.8)	9 (12.5)		
GA	1 (6.2)	6 (8.3)		
Interferon	1 (6.2)	3 (4.2)		
Other	0	7 (9.8)		
**ARR previous year pre-alemtuzumab** mean (SD)	1 (0.82)	0.72 (1.02)	0.30	0.27
**ARR over 2 years for alemtuzumab** mean (SD)	0.095 (0.25)	0.11 (0.27)	0.07	0.95
**Active lesions at baseline**, n (%)			0.61	0.019
0–2	8/15 (53.3)	52/64 (81.3)		
3+	7/15 (46.7)	12/64 (18.7)		
**MRI activity over 2 years for lemtrada**, n (%)	4 (25)	18 (25)	0.00	0.96
**Adverse events for alemtuzumab**, n (%)	8 (50)	26 (36.1)	0.28	0.26
**Lymphocyte baseline**, median (IQR)	2040 (1100–2490) [n = 14]	1790 (1460–2910) [n = 43]	0.24	0.62
**Lymphocyte at 24 months**, median (IQR)	1055 (775–1405) [n = 16]	1150 (810–1430) [n = 69]	0.095	0.83
**CD3 baseline**, median (IQR)	1638 (642–1861) [n = 8]	1097 (927–1560) [n = 10]	0.34	0.56
**CD3 at 24 months**, median (IQR)	500 (403–528) [n = 9]	550 (397–751) [n = 21]	0.52	0.28
**CD8 baseline**, median (IQR)	506 (186–634) [n = 11]	405 (287–553) [n = 31]	0.098	0.90
**CD8 at 24 months**, median (IQR)	203 (122–258) [n = 15]	195 (149–335) [n = 69]	0.40	0.43
**CD4 baseline**, median (IQR)	578 (250–1104) [n = 11]	810 (576–1330) [n = 31]	0.57	0.15
**CD4 at 24 months**, median (IQR)	210 (165–300) [n = 15]	251 (171–354) [n = 69]	0.28	0.43
**CD20 baseline**, median (IQR)	316 (125–368) [n = 10]	299 (177–450) [n = 29]	0.35	0.56
**CD20 at 24 months**, median (IQR)	255 (170–480) [n = 14]	189 (122–270) [n = 54]	0.55	0.11
**Follow-up**, median (IQR)	3.3 (2.5–4.4)	3.5 (3.0–4.2)	0.21	0.10
**N. of cycles**, n (%)			NC	0.11
1	1 (6.2)	3 (4.2)		
2	12 (75.0)	67 (93.0)		
3	3 (18.8)	2 (2.8)		

SMD, Standardized Mean Difference.

**Table 3:** Demographic and clinical characteristics associated with time to other treatment switch after alemtuzumab.

	Switched (n = 24)	Not Switched (n = 126)	HR (95% CI)	*p*-Value
**Age**, mean (SD), range	40.2 (9.5), 27–64	35.2 (10.4), 18–66	1.03 (0.99–1.07)	0.070
**Females**, n (%)	13 (54.2)	90 (71.4)	1.98 (0.89–4.43)	0.096
**Years from symptom onset**, median (IQR)	13.3 (6.8–18.4)	5.5 (2.2–11.4)	1.05 (1.01–1.10)	0.015
**Years from diagnosis**, median (IQR)	10.5 (3.8–14.8)	4.7 (1.7–9.1)	1.07 (1.01–1.13)	0.028
**Baseline EDSS**, median (IQR), range	5 (4–6), 1–7	2.5 (2–4), 0–8.5	1.37 (1.11–1.68)	0.003
**N. of previous DMTs**, median (range)	3 (0–6)	2 (0–6)		
**Previous DMTs**, n (%)				0.78
Naive	3 (12.5)	20 (15.9)		
Natalizumab	9 (37.5)	41 (32.5)		
Fingolimod	6 (25.0)	27 (21.4)		
Dimethyl-fumarate	2 (8.3)	18 (14.3)		
GA	3 (12.5)	6 (4.8)		
Interferon	1 (4.2)	6 (4.8)		
Other	0	8 (6.4)		
**ARR previous year pre-alemtuzumab**, mean (SD)	0.79 (0.78)	0.67 (0.94)	1.08 (0.73–1.58)	0.71
**ARR over 2 years for alemtuzumab**, mean (SD)	0.25 (0.53)	0.17 (0.49)	1.44 (0.71–2.91)	0.31
**Active lesions at baseline**, n (%)				
0–2	16/22 (72.7)	91/114 (79.8)	1.00 (ref)	
3+	6/22 (27.3)	23/114 (20.2)	1.62 (0.63–4.16)	0.31
**MRI activity over 2 years for alemtuzumab**, n (%)	9 (37.5)	26 (20.6)	1.91 (0.83–4.38)	0.13
**Adverse events for alemtuzumab**, n (%)	5 (20.8)	57 (45.2)	0.46 (0.17–1.23)	0.12
**Lymphocyte baseline**, median (IQR)	2370 (1500–2630) [n = 15]	1870 (1360–3240) [n = 95]	3.18 (1.01–10.01) *	0.048
**Lymphocyte at 24 months**, median (IQR)	855 (700–1395) [n = 24]	1145 (800–1390) [n = 118]	0.45 (0.19–1.05) *	0.066
**CD3 baseline**, median (IQR)	1736 (1170–1775) [n = 7]	927 (690–1560) [n = 45]	6.07 (0.72–50.97) *	0.097
**CD3 at 24 months**, median (IQR)	626 (466–740) [n = 4]	510 (386–626) [n = 70]	1.80 (0.54–5.95) *	0.44
**CD8 baseline**, median (IQR)	490 (405–624) [n = 15]	362 (270–553) [n = 73]	3.69 (1.04–13.13) *	0.044
**CD8 at 24 months**, median (IQR)	215 (122–279) [n = 24]	195 (140–310) [n = 117]	1.30 (0.57–2.94) *	0.53
**CD4 baseline**, median (IQR)	924 (705–1149) [n = 15]	598 (440–1239) [n = 73]	3.95 (1.11–14.10) *	0.034
**CD4 at 24 months**, median (IQR)	210 (165–329) [n = 24]	250 (146–323) [n = 117]	0.58 (0.25–1.33) *	0.20
**CD20 baseline**, median (IQR)	354 (120–552) [n = 15]	230 (150–416) [n = 61]	1.38 (0.47–4.06) *	0.56
**CD20 at 24 months**, median (IQR)	207 (74–259) [n = 17]	219 (143–323) [n = 104]	0.82 (0.31–2.12) *	0.68
**N. of cycles**, n (%)				
1	3 (12.5)	9 (7.1)		
2	20 (83.3)	112 (88.9)		
3	1 (4.2)	5 (4.0)		

* Hazard ratio reported for categorized >median value vs. ≤ median value.

## Data Availability

Data are available on request.

## Supplementary Materials

The following supporting information can be downloaded at: https://www.mdpi.com/article/10.3390/jcm12051768/s1, Table S1: Clinical characteristics in patients with and without clinical or MRI activity after 24 months from treatment start in the whole cohort.
