# Supplementary material for: Alemtuzumab-Related Lymphocyte Subset Dynamics and Disease Activity or Autoimmune Adverse Events: Real-World Evidence
Source: J Clin Med. 2023 Feb 22;12(5):1768. doi: 10.3390/jcm12051768 (PMC10002781; doi:10.3390/jcm12051768)
Supplement: Supplementary file 1 [file jcm-12-01768-s001.zip › jcm-2189927-supplementary.pdf]

Table S1. Clinical characteristics in patients with and without clinical or MRI activity after 24 months from treatment start in the whole cohort.

	Clinical or MRI Activity After 24 Months (n = 16)	No Clinical Activity after 24 Months (n = 134)	SMD	*p*-Value
**Age**, mean (SD), range	32.5 (9.4), 18–56	36.2 (10.5), 18–66	0.39	0.13
**Females**, n (%)	7 (43.7)	96 (71.6)	0.58	0.032
**Years from symptom onset**, median (IQR)	6.3 (2.5–11.1)	6.8 (2.2–12.7)	0.053	0.66
**Years from diagnosis**, median (IQR)	4.7 (2.1–10.3)	5.2 (1.9–10.3)	0.034	0.79
**Baseline EDSS**, median (IQR), range	2.5 (1–6), 1–7	3 (2–5), 0–8.5	0.066	0.59
**Wash-out before lemtrada**, median (IQR)	1.5 (1.1–2.4)	2.1 (1.3–3.5)	0.074	0.96
**N. of previous DMTs**, median (range)	2 (0–6)	2 (0–6)	0.15	0.77
**Previous DMTs**, n (%)			NC	0.93
Naive	2 (12.5)	21 (15.7)		
Natalizumab	4 (25)	46 (34.3)		
Fingolimod	5 (31.3)	28 (20.9)		
Dimethyl-fumarate	3 (18.8)	17 (12.7)		
GA	1 (6.2)	8 (6.0)		
Interferon	1 (6.2)	6 (4.5)		
Other	0	8 (5.9)		
**ARR previous year pre-alemtuzumab,** mean (SD)	1 (0.82)	0.65 (0.92)	0.40	0.19
**ARR over 2 years for alemtuzumab,** mean (SD)	0.095 (0.29)	0.11 (0.26)	0.02	0.96
**Active lesions at baseline**, n (%)			0.62	0.018
0–2	8/15 (53.3)	99/121 (81.8)		
3+	7/15 (46.7)	22/121 (18.2)		
**MRI activity over 2 years for alemtuzumab**, n (%)	4 (25)	31 (23.1)	0.043	0.93
**Adverse events fir alemtuzumab**, n (%)	8 (50)	54 (40.3)	0.19	0.30
**Lymphocyte baseline**, median (IQR)	2040 (1100–2490) [n = 14]	1995 (1426–3245) [n = 96]	0.31	0.44
**Lymphocyte at 24 months**, median (IQR)	1055 (775–1405) [n = 16]	1075 (780–1390) [n = 126]	0.024	0.81
**CD3 baseline**, median (IQR)	1638 (642–1861) [n = 8]	974 (740–1560) [n = 45]	0.26	0.66
**CD3 at 24 months**, median (IQR)	500 (403–528) [n = 9]	520 (389–684) [n = 65]	0.27	0.49
**CD8 baseline**, median (IQR)	506 (186–634) [n = 11]	380 (274–553) [n = 78]	0.074	0.98
**CD8 at 24 months**, median (IQR)	203 (122–258) [n = 15]	200 (134–314) [n = 126]	0.33	0.49
**CD4 baseline**, median (IQR)	578 (250–1104) [n = 11]	704 (440–1310) [n = 78]	0.41	0.32
**CD4 at 24 months**, median (IQR)	210 (165–300) [n = 15]	245 (146–329) [n = 126]	0.14	0.75
**CD20 baseline**, median (IQR)	316 (125–368) [n = 10]	236 (140–434) [n = 67]	0.20	0.90
**CD20 at 24 months**, median (IQR)	255 (170–480) [n = 14]	210 (133–304) [n = 107]	0.48	0.16
**Follow-up**, median (IQR)	3.3 (2.5–4.4)	2.7 (1.9–3.6)	0.65	0.48
**N. of cycles**, n (%)			NC	0.060
1	1 (6.3)	11 (8.2)		
2	12 (75)	120 (89.6)		
3	3 (18.8)	3 (2.2)		

SMD, Standardized Mean Difference.
